# Dental research related to COVID-19 in Brazil: research presented at the 38^th^ *SBPqO* Meeting

**DOI:** 10.1590/1807-3107bor-2024.vol38.0035

**Published:** 2024-05-13

**Authors:** Luisa GATTI-REIS, Alice Corrêa SILVA-SOUSA, Isabela Almeida PORDEUS, Saul Martins PAIVA, Flávio Freitas MATTOS

**Affiliations:** (a) Universidade Federal de Minas Gerais – UFMG, School of Dentistry, Department of Pediatric Dentistry, Belo Horizonte, MG, Brazil.; (b) Universidade de São Paulo – USP, School of Dentistry, Department of Restorative Dentistry, São Paulo, SP, Brazil.; (c) Universidade Federal de Minas Gerais – UFMG, School of Dentistry, Department of Social and Preventive Dentistry, Belo Horizonte, MG, Brazil.

**Keywords:** Dentistry, Dental Research, COVID-19, Pandemics

## Abstract

The aim of this study was to identify and describe the characteristics of coronavirus (COVID-19)-disease related dental research in Brazil presented at the 38th Annual Meeting of the Brazilian Division of the International Association for Dental Research (SBPqO). A search was carried out in the proceedings of the meeting to retrieve all abstracts. Those containing the term “COVID-19” in titles, abstracts, or keywords, and/or those of which the scope approached a COVID-19-related topic were included. The variables extracted from abstracts were: presenter category, field of study, design, data collection method, population, affiliation, and authors’ gender. Descriptive and inferential statistics were used, with a significance level of α = 0.05. The search retrieved 185 abstracts, 5 did not meet study eligibility criteria and were excluded. COVID-19-related research was presented by either aspiring/associate members (67.8%) or beginner members (32.2%). Data collection methods were predominantly digitally mediated (65%), followed by secondary data use (25%), and in-person data collection (7.2%). Irrespective of the role of authorship, there were a ratio of two female authors to each male. Among the last authors, the ratio was three females to each male. Female lead authors more frequently came from the Southeast region (71.8%; p = 0.470). There was an association between presenter category and study design (p = 0.012), clinical and epidemiological studies were more concentrated among experienced presenters. In conclusion, female dental researchers affiliated to southeastern institutions approached the topic of pandemic more frequently than male colleagues. The use of digital technology for data collection may have long-lasting impacts on the teaching and publication of dental research.

## Introduction

In March 2020 the World Health Organization declared the outbreak of coronavirus disease (COVID-19), a pandemic caused by severe acute respiratory syndrome coronavírus, SARS-Cov-2.^1^Since then, it has led to significant and long-lasting impacts across different domains, ranging from social to economic, and healthcare systems, including dental practice.^2^ Across the Globe, social distancing measures involving the closure/restrictive operation of schools, universities, research centers, and health services, were implemented to mitigate the spread of infection. ^2-4^

In Dentistry, there was a limited operation of dental clinics due to fear of cross-infection, scarce personal protective equipment, and temporary restriction of dental care services to emergency care.^2^ Changes in dental practice included avoidance of procedures that generated bioaerosols, as well as the establishment of new biosafety protocols.^4,5^The aforementioned challenges also impacted dental research, which required extra expenses due to new safety requirements, time, resources, and challenges to data collection.^6^ In 2023 there are COVID-19 vaccines, however, the pandemic is still ongoing with outbreaks of infection caused by variants of interest emerging across the world, with impact on the provision of dental care and research.^7-9^

Recent evidence has highlighted Brazilian contribution to dental research: Brazil has ranked second in the number of international publications in Dentistry for over fifteen years and ranked second in citations in 2017.^10^ Held in Brazil, the Annual Meeting of the Brazilian Division of the International Association for Dental Research - Sociedade Brasileira de Pesquisa Odontológica (SBPqO), is the largest dental research conference in Latin America. During the meeting, researchers presented their original work on all subjects of dental science, which were evaluated by senior members of the SBPqO, and later on, the abstracts were published as proceedings of the meeting.

Research presented at the SBPqO meeting has occupied a position of innovation and relevance. Nevertheless, little is known about COVID-19-related research presented at the meeting. In view of the foregoing, the aim of this study was to identify and describe the characteristics of COVID-19-related dental research in Brazil presented at the 38^th^ Annual Meeting of the Brazilian Division of the International Association for Dental Research - SBPqO.

## Methods

This observational cross-sectional study was reported following the Strengthening the Reporting of Observational studies in Epidemiology (STROBE) statement checklist.^11^

### Study design and eligibility criteria

A bibliometric search was carried out in the first trimester of 2022 to retrieve the abstracts of research presented at the 38th Meeting of the Brazilian Division of the IADR (2021) and published as proceedings of the meeting in the Brazilian Oral Research (https://www.sbpqo.org.br/hotsite2021/bor-v035-sbpqo-book_2021.pdf). Two authors (LGR and ACSS) independently selected the abstracts in two steps. In the first step, both authors selected abstracts containing the term “COVID-19” in the title and keywords. The abstracts with titles that appeared to fulfill the eligibility criteria were screened in the second step and included in this study. Abstracts authored by researchers affiliated to non-Brazilian institutions were excluded. Any disagreements were solved by consensus with a group of four researchers with experience in Epidemiological studies.

### Variables of interest

**Figure 1:**
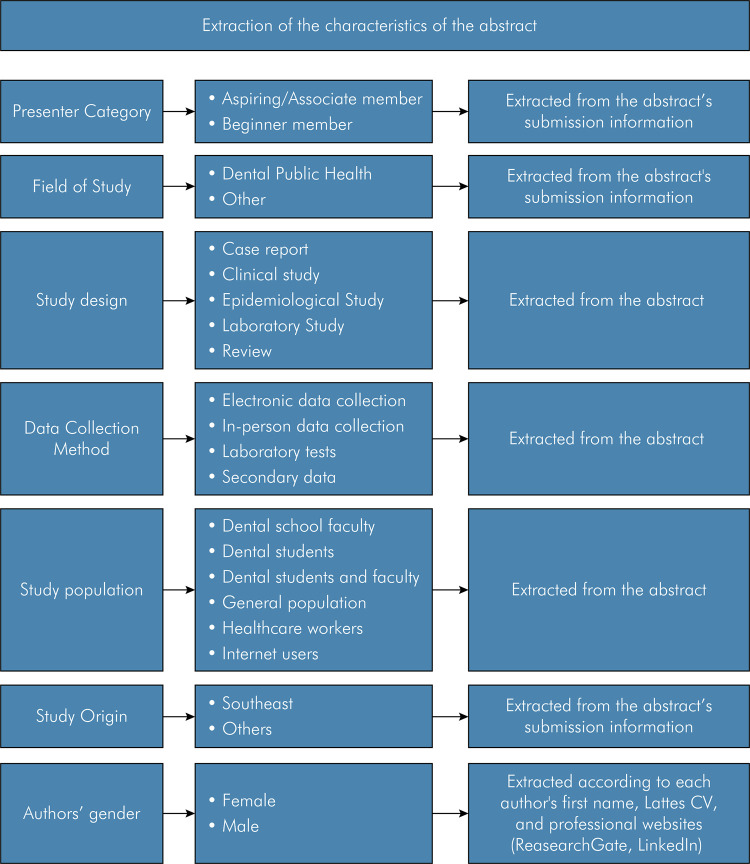
Data extraction of abstract characteristics and sources

In this study, the variables study origin, presenter category, author’s gender, role of authorship (first/last authorship), and study design were collected. Data extraction of study origin and presenter category were derived from the abstract submission information. The authors’ gender (male or female) was classified individually. At first, authors’ first names were evaluated, and when necessary, a manual search were carried out using Lattes platform to check their *Curriculum Vitae* (https://lattes.cnpq.br), and professional websites (ResearchGate, LinkedIn). The study design was extracted from reading the abstracts (Figure 1).

The following bibliometrics parameters were extracted from each abstract selected: presenter category (aspiring/associate or beginner member); field of study, classified as dental public health, or others; number of authors per publication; study design, categorized as case report, clinical, epidemiological, laboratory studies, and review; data collection method, categorized as electronic data collection, and in-person data collection, laboratory tests, secondary data; study population, categorized as dental school, dental students, and general population, healthcare workers, internet users; study origin, according to the institutional affiliation of the first author seen in the abstract publication; total number of authors (total female and male authors), first, intermediate, and last author’s gender.

Data extraction was carried out independently by two researchers and data were double-checked for accuracy (LGR and ACSS). Any discrepancy was resolved by re-evaluating the original abstract. A panel of four researchers with vast experience in Epidemiological studies analyzed the characteristics of the abstracts and solved discrepancies by consensus.

### Statistical Analysis

Statistical analyses were performed with use of the Statistical Package for the Social Sciences (*SPSS for Mac*, version 25.0; IBM Corp., Armonk, N.Y, USA) software program. Descriptive statistics were performed and results were expressed in absolute and relative frequencies. To assess the association between lead and senior authors’ gender and study origin (exposure variable), Pearson’s chi-square test was used. The same test was used to assess the association between study design and presenter category (exposure variable). In the analyses, a significancy level of α = 0.05 was considered.

## Results

**Figure 2:**
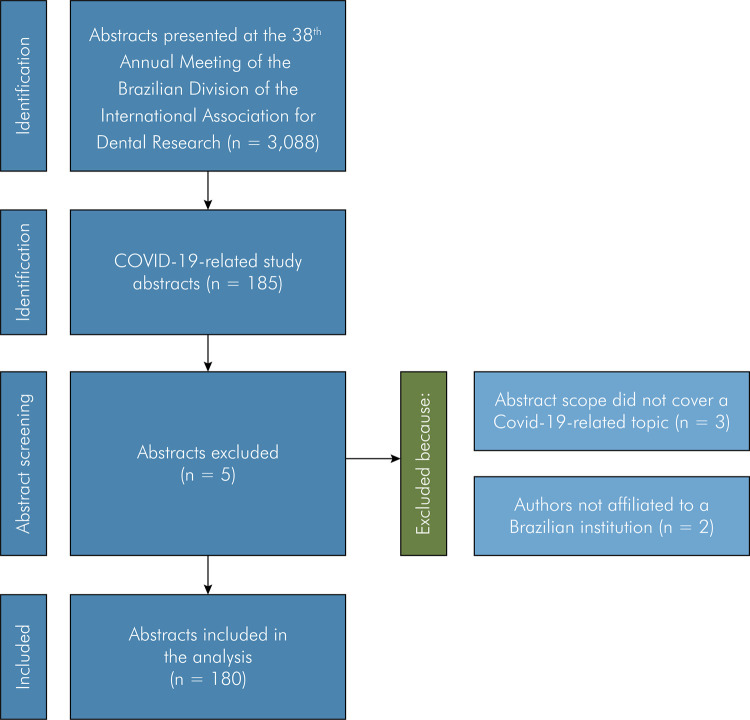
Abstract selection flow diagram.

The search strategy retrieved 185 study abstracts presented at the 38^th^ Annual Meeting of the Brazilian Division of the International Association for Dental Research in 2021. After initial screening, 5 abstracts did not match the eligibility criteria and were excluded (Figure 2). After exclusions, 180 abstracts were included.

The characteristics of all the abstracts included are presented in Table 1. COVID-19-related research articles were presented by aspiring/associate members (67.8%) and beginner members (32.2%). The most frequent field of study was Dental Public Health (n=101, 56.1%), in comparison with others (Pediatric Dentistry, Infection control/Microbiology/Immunology, Periodontology, Orthodontics, Stomatology, Occlusion, Radiology, Endodontics, Dental materials, Prosthodontics, Restorative dentistry, Physiology/Biochemistry/Pharmacology).

**Table 1:** Frequencies of characteristics of the publications

Characteristics	n (%)
Presentation category	
Aspiring/ Associate member	122 (67.8)
Beginner member	58 (32.2)
Field of study	
Dental Public Health	101 (56.1)
Others*	79 (43.9)
Number of authors per publication	
2-4	38 (21.1)
5-6	57 (31.7)
7-8	85 (47.2)
Study design	
Case report	14 (7.8)
Clinical Study	2 (1.1)
Epidemiological Study	133 (73.9)
Laboratory study	4 (2.2)
Review	27 (15.0)
Data Collection Method	
Electronic data collection	117 (65.0)
In-person data collection	13 (7.2)
Laboratory tests	5 (2.8)
Secondary data	45 (25.0)
Study population	
Dental school	4 (2.7)
Dental students	37 (24.8)
Dental students and School	8 (5.4)
General population	66 (44.3)
Healthcare workers	30 (20.1)
Internet users	4 (2.7)
Others**	31 (17.2)
Study Origin	
Midwest	22 (12.2)
North	3 (1.7)
Northeast	24 (13.3)
South	28 (15.6)
Southeast	103 (57.2)
Total number of authors (first, last, intermediate)	
Total female authors	758 (68.8)
Total male authors	344 (31.2)
First author gender	
Female	133 (73.9)
Male	47 (26.1)
Intermediate authors’ gender	
Female	487 (65.6)
Male	255 (34.4)
Last author gender	
Female	138 (76.7)
Male	42 (23.3)

* Pediatric Dentistry, Infection control/Microbiology/Immunology, Periodontology, Orthodontics, Stomatology, Occlusion, Radiology, Endodontics, Dental materials, Prosthodontics, Restorative dentistry, Physiology/Biochemistry/Pharmacology.

** Laboratory and review studies.

Abstracts were written by 1,102 researchers in total. Studies written by 7-8 authors (47.2%) or 5-6 authors (31.7%) were more frequent than those with 2-4 authors (21.1%). Epidemiological studies (73.9%), reviews (15.0%), and case reports (7.8%) were the most common study designs. The data collection methods were predominantly electronic (65%), followed by secondary data (25%) and in-person data collection (7.2%). The majority of studies were conducted with samples of the general population (44%), and were more frequently presented by authors affiliated to institutions located in the Southeast region of Brazil (57.2%). Evaluation of the authors’ gender showed that abstracts were more frequently written by females (68.8%) than males (31.2%). Irrespective of the role of authorship, there was a ratio of 2 female authors to each male author. Among senior authors, the ratio was 3 females to each male.

The results of the bivariate analyses are displayed in Tables 2 and 3. Female lead authors more frequently came from the Southeast (71.8%; p=0.470). Among senior authors, 84.5% were females who originated from the Southeast (p=0.004). There was an association between presenter category and study design (p=0.012), with clinical and epidemiological studies being more concentrated among more experienced presenters.

## Discussion

This study identified and analyzed the main characteristics of COVID-19-related dental research in Brazil presented at the 38th Annual Meeting of the Brazilian Division of the IADR in 2021. The COVID-19-related research articles presented at the meeting were more frequently shown to be Epidemiological studies that used electronic data collection and were presented by authors who were aspiring/ associate members, women, and affiliated to Southeastern institutions.

Brazil occupies a prominent role in dental research worldwide, and although COVID-19 vaccines are now available, the pandemic persists with outbreaks of variants of interest that continue to cause concern and impact on dental practice and research. Hence, it is noteworthy the authors emphasize that to advance evidence-based dental care, there is an urgent call to gain full understanding of what is being developed and by whom.

In the organization of Brazilian dental research, the beginner member category is composed of undergraduate dental students who are often being introduced to research, whereas members of the aspiring and associate categories are graduate students and senior researchers, respectively. Young scientists may possibly not have therefore review studies are favored rather than clinical and epidemiological research.

During the COVID-19 pandemic, to mitigate infection from the virus, access to places where traditional data collection is carried out in dental research, such as research centers, dental offices, hospitals, schools, was restricted. At this time, researchers saw the need to adapt research procedures and resorted to electronic data collection methods. ^6^Although these methods were not new to dental research; the advent of the pandemic saw a rapid increase in their use. The widespread use of electronic data collection can be seen in two ways. It was needed to provide dental practitioners and stakeholders with the necessary data to adapt the profession to the challenging new reality and data needed to be collected and analyzed quickly. However, the rush for evidence has often been accompanied by a possible downside, for instance, the use of non-validated questionnaires might have a relevant impact on the internal validity of the results obtained. Therefore, some strategies have been suggested for those conducting remote data collection - sending SMS reminders, for instance, has been highlighted as a strategy to improve the participation of volunteers, possibly leading to higher rates of response to a given instrument.^12^

The use of digital technology for data collection during this period may have long-lasting impacts on dental research, publication, and education. With regard to the latter, the closure of teaching institutions saw an urgent need to shift to online learning. Professors and students across the globe had to adapt to a situation that would become known as “the new normal”. In Dental education, a shift towards non-traditional teaching methods such as problem-based, active learning, and use of electronic methods has been developing over the course of the past few years.^13,14^ During the pandemic, from one aspect there was a heightened interest in dental education and a shift to these teaching strategies across the globe.^15^From another aspect, however, the recommendation has been that dental education strategies should be tailored to students’ needs and characteristics. A present challenge in teaching Generation Z is the possibility that they might favor in-person communication over electronic.^15^

In our analyses, the majority of studies were presented by authors affiliated to Southeast institutions (57.2%). The unequal representation relative to author affiliation reflects the distribution of undergraduate and graduate Dentistry courses in Brazil. Although still present, the unequal distribution of Dentistry courses has narrowed over the last decades. In 1991, the majority of undergraduate institutions were located in the Southeast (58%), with only 2% in the North region. Despite the drop in representation of Southeastern institutions to 36% by 2020, regional inequalities persist.^16^ In graduate dental education, institutions are more frequently located in the Southeast. Back in 2009, out of 95 programs, two-thirds were in the South;^17^ while in 2019, out of 102 programs, 60 (58.82) were in the Southeast.^18^ It can be inferred that the unequal regional distribution of author affiliations in our study reflects these pervasive regional inequalities, at both undergraduate and graduate levels, in dental education in Brazil.

During the COVID-19 pandemic, restrictive measures highlighted pre-existing inequalities in Science and Academics, and dental research appeared to be no exception. Reports have indicated negative consequences that disproportionately affect female researchers, in their early career stages, with intersecting identities, and those from countries deeply affected by COVID-19.^3,4,6,8^ In contrast, our analysis demonstrated that the majority of first and last roles in authorship were women. This distribution can be observed in Brazilian Dentistry, in which women are the majority of registered dentists (61.0%) and dental researchers (55.4%).^19^

Based on the literature reviewed, we found no paper with similar aims/methods. Moreover, considering that the SBPqO congress is the largest dental research meeting held in Latin-America and covers the full scope of Dentistry, it has provided a bird’s eye view of knowledge production in Brazilian COVID-19-related dental research. Furthermore, the authors sought to assess characteristics of the abstracts included, such as the authors’ gender, affiliation, and demographic data, which is a novelty.

However, the study was not without its limitations. Firstly, the cross-sectional design provided a snapshot of the COVID-19 research landscape in Brazil during the first two years of the pandemic (2020-2021), by presentation of abstracts at SBPqO meeting. Secondly, this study sought to provide an overview of the characteristics of COVID-19-related dental research presented at the conference meeting - in the absence of a control group (abstracts presented in pre-pandemic years), it was not possible to assess the impact of the COVID-19 pandemic on dental research presented at the conference. Further studies are needed to assess dental research in Brazil by comparing data from the periods before and during the pandemic. This would allow for a more comprehensive analysis of the impact of the COVID-19 pandemic on dental research and implications for the future. Moreover, gender is a non-binary variable; however, it was considered in this study as such (male/female) since limited time and resources did not allow the authors to contact each researcher directly to establish their gender identity. Therefore, future studies should focus on the assessment of the pandemic-related dental research in Brazil, in addition to considering gender inequality visualized through intersectional lenses.^20^

## Conclusions

The study assessed COVID-19-related dental research in Brazil presented at the 38th Annual Meeting of the Brazilian Division of the International Association for Dental Research in 2021 (IADR) – SBPqO. Female dental researchers affiliated to southeastern institutions approached the pandemic topic more frequently than their male colleagues.

**Table 2:** Distribution of the first and last authors’ gender, according to the category (N=180).

Variable/Category	First author gender				Last author gender			

	Total *n* (%)	Female *n* (%)	Male *n* (%)	*p*-value*	Total *n* (%)	Female *n* (%)	Male *n* (%)	*p*-value*
Study origin				0.470				0.004
Southeast	103 (57.2)	74 (71.8)	29 (28.2)		103 (57.2)	87 (84.5)	16 (15.5)	
Other	77 (42.8)	59 (76.6)	18 (23.4)		77 (42.8)	51 (66.2)	26 (33.8)	

*Pearson Chi-Square test.

**Table 3:** Distribution of the study design, according to the independent variable (N=180).

Variable/Category	Total	Study design					

		Case report	Clinical	Epidemiological	Laboratory	Review	*p*-value*
Presentation category							0.012
Aspiring/associate member	122 (67.8)	10 (8.2)	2(1.6)	95 (77.9)	4 (3.3)	11 (9.0)	
Beginner member	58 (32.2)	4 (6.9)	0 (0.0)	38 (65.5)	0 (0.0)	16 (27.6)	

*Pearson Chi-Square test.
